# Hepatitis E virus and blood transfusion safety

**DOI:** 10.1017/S0950268820001429

**Published:** 2020-06-29

**Authors:** Hao Bi, Ru Yang, Chunchen Wu, Jianbo Xia

**Affiliations:** 1 Department of Laboratory Medicine, Maternal and Child Health Hospital of Hubei Province, Wuhan 430070, China; 2 Department of Blood Transfusion Research, Wuhan Blood Centre, Wuhan 430030, China

**Keywords:** Blood donors, blood safety, HEV, transfusion, TT-HEV

## Abstract

While the majority of worldwide hepatitis E viral (HEV) infections that occur in people are from contaminated water or food sources, there has also been a steadily rising number of reported cases of transfusion-transmitted HEV (TT-HEV) in blood donation recipients. For most, HEV infection is acute, self-limiting and asymptomatic. However, patients that are immunocompromised, especially transplant patients, are at much higher risk for developing chronic infections, which can progress to cirrhosis and liver failure, along with overall increased mortality. Because of the rising trend of HEV serological prevalence among the global population, and the fact that TT-HEV infection can cause serious clinical consequences among those patients most at need for blood donation, the need for screening for TT-HEV has been gaining in prominence as an important public health concern for both developing and developed countries. In the review, we summarise evidence for and notable cases of TT-HEV infections, the various aspects of HEV screening protocols and recent trends in the implementation of TT-HEV broad-based blood screening programmes.

## Introduction

Hepatitis E (HE) is considered to be the most common cause of acute hepatitis worldwide. Initially, it was discovered through retrospective studies of cases of hepatitis in India [1]. The disease-causing pathogen, hepatitis E virus (HEV), was subsequently confirmed through voluntary ingestion of infectious material by a Russian researcher, which was isolated and visualised from his own stool samples following his own course of illness. HEV virus is a small, non-enveloped, positive-template, single-stranded RNA virus, encased within an icosahedral capsid of between 27 and 34 nm in size [2]. HEV infection typically follows a fairly routine clinical course, consisting of an incubation period of 2–6 weeks, followed by a few weeks of pronounced detectable viraemia in both serum and stool along with corresponding symptoms of hepatitis (abdominal pain, vomiting, hepatomegaly, jaundice, etc.). Resolution coincides with a typical immune response of IgM antibody production that precedes the appearance of more durable IgG antibodies. HE disease course is usually self-limiting; however, immunocompromised individuals can develop persistent chronic infections and are at risk for more serious hepatic complications. Furthermore, pregnancy can pose unique risks when coinciding with HEV infection, including higher rates of premature births, stillbirths and mortality. Worldwide, HEV infection is mainly prevalent in Asia, Africa and Latin America, and can occur either endemically or sporadically, though it is relatively uncommon outside developing countries [3]. Among those populations at risk, the incidence of HE is higher among youth than adults, with greater infection rates in men relative to women [4, 5]. Transmission routes and geographic distribution of HEV typically stratify by genotypes [3]. The most predominant forms of infections, types 1 and 2, occur via contaminated water sources through faecal–oral routes, and are usually found in developing countries. In contrast, type 3 and 4 HEV infections, the predominant types found in the developed world, are thought to only be contracted zoonotically, often the result from the consumption of contaminated sources of farm animal-derived food products.

While these viral reservoirs supply the bulk of most HEV infections, HEV can also be transmitted during blood transfusions [6]. Since the first reported transfusion-transmitted HEV (TT-HEV) infection case in 2004 [7], more cases have successively been reported in Japan [8, 9], France [10–14], the UK [15, 16], Germany [17] and Spain [18]. TT-HEV infections are typically asymptomatic, similar to most cases of HEV infections that occur in developed countries, and as such have historically been neglected [16]. Nevertheless, given the rising trend of HEV serological prevalence among the general population (and by extension prospective blood donors), and that TT-HEV infection can cause serious clinical consequences especially among immunocompromised and pregnant patients, TT-HEV incidence has been gradually receiving increased attention as an important public health problem in both developing and developed countries.

## Evidence of transmission of HE through blood transfusion

Previous studies have shown a higher rate of anti-HEV immunoglobulin (Ig)G positivity in patients who had received multiple transfusions *vs.* those in the healthy control group [19–22], indirectly indicating that transfusions, especially multiple transfusions, may be a route for HEV transmission. Additionally, the transfusion of human blood containing HEV into a macaque caused HEV infection, also suggesting HEV may be transmitted through blood [23]. However, the first direct evidence for TT-HEV infection from human-to-human blood transfusion was reported in Japan [7], with cases successively reported in Japan [8, 9, 24], France [10, 11] and the UK [15]. In all of these cases, the HEV genomic sequence from the blood donor and patient matched identically, thus confirming that the origin of the HEV infections was from the blood donor and had been transmitted to the patient from transfusion (see Box 1 for further details).

Box 1 TT-HEV case study details

Since the initial discovery of the HEV virus, the bulk of most HEV infections that were diagnosed among the general population occur from water/food-borne contamination. Nevertheless, a small but significant proportion of cases of HEV transmission are known to occur from blood transfusions in patients that are uniquely susceptible to some of the worst consequences of the disease. While several lines of evidence pointed to the potential for HEV infections deriving from contaminated blood supplies [7–11, 15, 24], the first confirmatory evidence of direct donor-to-recipient transmission of HEV, as verified by RNA sequence analysis, was reported in a patient in Hokkaido Japan in 2004 [7]. Since then, there have been several notable cases of TT-HEV infections reported worldwide, underscoring the need for more prevalent testing procedures in blood banks. Details from these published case reports of definite TT-HEV infections are summarised below:

- The first bona fide TT-HEV infection was reported in Hokkaido, Japan, in 2004. After the male patient developed acute hepatitis after a blood transfusion during his cardiac surgery, one of the blood donors was found to be infected with asymptomatic HEV. Although the donor had normal alanine aminotransferase (ALT) activity and no travel history, he tested positive for HEV RNA levels. RNA sequencing of both the patient and donor showed they had identical genotype 4 HEV viral genomic sequences [7].
- In 2007, a patient with a T-cell lymphoma in Japan was diagnosed with an HEV infection lasting for 6 months. The patient received erythrocytes (RBCs) from 44 donors and platelets from 40 donors during chemotherapy. Subsequent investigation found that one of the RBC products was HEV type 3-positive [8].
- In 2008, a patient in Japan with Hodgkin's lymphoma was infected with HEV type 4 after an autologous bone marrow transplantation and adjuvant transfusion therapy post-chemotherapy. Further studies showed that one blood donor had eaten pork at a barbecue with 13 relatives 23 days before his blood donation, with the donor's father having died from acute hepatitis E and six of his relatives testing positive for anti-HEV antibodies [9].
- In 2004, another research group in Japan found that four patients having received ongoing haemodialysis tested positive for anti-HEV antibodies, and one of them was confirmed to be infected with HEV type 3 from a blood transfusion [24].
- In 2006, a potential source for multiple TT-HEV infections was reported in the UK. The donor did not have contact with pigs or pork, and had no travel history. He had no symptoms when he donated blood, but had transient influenza symptoms and jaundice after the blood donation. Cancer patients receiving RBCs from the donor during chemotherapy were confirmed to be infected with HEV type 3 [15].
- In 2007, French doctors reported that a 7-year-old boy with kidney cancer, who had received concentrated RBCs and platelets from 22 blood donors after his chemotherapy, was confirmed to be infected with HEV. The virus was found to have come from an asymptomatic donor of the HEV type 3f [10].
- Another case from France was reported in 2012, where an 81-year-old man with heart disease and autoimmune thrombocytopaenia developed persistent liver damage after treatment for his haematologic disorders. After 3 months of misdiagnosis (drug-induced hepatitis, autoimmune hepatitis and other hepatitis-causing viruses were all eventually ruled out), he was finally correctly diagnosed as having contracted hepatitis from a TT-HEV infection from an HEV type 3f [11].

## Detection of HEV infection

HEV can be detected either indirectly, by assaying serological anti-HEV antibodies, or directly, by measuring HEV RNA or HEV antigen levels in the blood. Each approach has a limited time frame following diagnosis. The window for IgM detection before seroconversion is 2–6 weeks following disease onset; at this point, increasing alanine aminotransferase (ALT) activity can be detected, which lasts for 6–9 months. The appearance of IgG is usually delayed compared to IgM, but can last for many years. Both HEV RNA and HEV capsid antigen levels peak early in the disease and last ~4 weeks.

### Serological antibody diagnosis

Commercial reagents include a traditional microplate ELISA method and rapid immunochromatography. Anti-HEV IgM is a marker for acute infection while anti-HEV IgG is a marker for post-infection. The limit of detection (LoD) for most commercial anti-HEV IgG is in the range of 0.25–2.5 IU/ml, though the more sensitive detection commercial reagents (LoD: 0.25 IU/ml) are often used for studying HEV epidemiology.

### Serological antigen diagnosis

HEV viraemia can also be diagnosed directly through an ELISA-based capture assay of HEV capsid antigens. It has been reported that the specificity of antigen detection is high, but the sensitivity is poor, and the detection limit is 800–80 000 IU/ml [25]. While HEV RNA detection is far more sensitive and considered the ‘gold-standard’, antigen capture is simpler, cheaper and faster, and would be suitable for laboratories that lack other molecular diagnostic equipment.

### RNA detection and identification

Assaying HEV RNA directly, either from blood, faecal or other bodily fluid samples, is considered to be the ‘gold-standard’ for molecular diagnosis of HEV infection. HEV RNA is detected and quantified using a nucleic acid test (NAT), which involves isolation of RNA followed by amplification via primer-mediated enzyme extension. Protocols vary in clinical setups, but generally HEV RNA detection employs isothermal, single-step nucleic acid amplification technologies, such as transcription-mediated amplification (TMA) or reverse-transcription loop-mediated isothermal amplification to great success [26]. TMA, in particular, is well suited for high-throughput detection of HEV RNA from multiple serum samples on a fully automated commercialised platform [27]. These technologies are highly sensitive and robust, with detection limits in the range of 7–80 IU/ml, and, given such, can be extended beyond clinical diagnosis to more broad blood screening protocols. The primers used in diagnosis are designed to target the conservative genomic regions of all the different HEV genotypes, usually flanking the viral gene open reading frame 3 (ORF3). However, identification of HEV RNA can also be achieved by sequencing other conserved regions of the HEV genome, such as ORF2 or OFR1, and are often used to identify particular HEV genotypes/subtypes or to track the source of infection by phylogenetic analysis.

## Serological viraemic prevalence of HEV among global blood donors

While HEV is not routinely screened during blood donation in most countries unlike other viral pathogens, there have been many prospective studies that have been conducted looking for markers of HEV infection in serum samples from potential blood donors, so as to assess the local risk for TT-HEV infection. The rates for various markers of HEV infection, namely anti-HEV IgG, anti-HEV IgM and HEV RNA positivity, are summarised in Tables 1–3, respectively. Broadly speaking, the overall rates of anti-HEV IgG reactivity among blood donors in Europe ranged from 4.7% to 52.5%, in Australia 6.0%, in central Asia from 14.3% to 21.48% and in the USA 16.0% [28]. These define three levels of prevalence: low (anti-HEV IgG <10%), medium (anti-HEV IgG: 10–20%) and high (anti-HEV IgG >20%). Taken as a whole, these results found that countries with a high rate anti-HEV IgG reactivity, such as France, Germany and the Netherlands, had a correspondingly higher prevalence of viraemia when compared to countries with lower seroreactivities (compare Tables 1 and 3). In these same countries, however, the rates of detectable HEV viraemia varied and is likely due to the differing NAT strategies adopted by each group: this can dramatically impact detection limits, false-positive and false-negative rates, statistical power, whether sampling was pooled or based on individual donations, etc. For example, in France, differences in rates of viraemia were observed when a pool of 96 samples was initially adopted (a rate of 1/2218 donors) [29] than when compared to when an individual test mode was used (a rate of 1/744 donors) [30] for similar regions. These studies also collectively found particular geographic distributions for TT-HEV genotypes: HEV type 1 was found only in Asia and North Africa; HEV type 2 was found only in Mexico and South Africa; HEV type 3 was found almost everywhere, in North and South America, Europe and Asia; while type 4 was only reported for donors in Asia.

**Table 1.** Rates of anti-HEV IgG positivity among blood donors worldwide

Low and medium prevalence regions	IgG-positive rate （%）	Reference	High-prevalence countries	IgG-positive rate （%）	Reference
England	12	[31]	The Netherlands	20.9–31.0	[32–34]
Scotland	4.7	[35]	France	22.4–23.6	[29, 36]
Ireland	5.3	[37]	Germany	29.5	[38]
Serbia	15	[39]	Poland	43.5	[40]
Switzerland	4.9	[41]	China	29.2	[42]
Austria	13.5	[43]			
Spain	19.9	[44]			
Spain	8.7–17.4	[45, 46]			
USA	10.7–12.3	[47, 48]			
Australia	6.0	[49]			
New Zealand	4.0–9.7	[50, 51]			
Fiji	2.0	[52]			
Saudi Arabia	18.7	[53]			
India	17.70	[54]

**Table 2.** Rates of anti-HEV IgM positivity among blood donors worldwide.

Region	Year	Sample size	Positive rate（%）	Assay used	Reference
USA	2015	5040	0.67	Wantai ELISA	[48]
			2.90	DSI ELISA	
			1.85	MP Biomedical ELISA	
Denmark	2008	491	20.6	In-house ELISA	[55]
Denmark	2015	504	10.7	In-house ELISA	[56]
Denmark	2015	504	19.8	Wantai ELISA	[56]
Sweden	2006	108	9.3	Abbott ELISA	[57]
Norway	2016	1200	14	In-house ELISA	[58]
England	2008	500	16–25	Genelabs ELISA	[59]
Scotland	2013	1559	4.7	Wantai ELISA	[35]
Wales	2011	262	10	Wantai ELISA	[31]
The Netherlands	2011	1275	0.4	Abbott ELISA	[32]
Austria	2015	1203	13.3	Wantai ELSA	[43]
Germany	2012	336	5.94	MP Biomedical ELISA confirmed by Mikrogen immunoblot	[38]
Germany	2013	1019	6.8	Mikrogen ELISA confirmed by Mikrogen immunoblot	[60, 61]
Germany	2011	116	15.5	Mikrogen immunoblot	[61]
France	2007	1998	3.2	Genelabs ELISA	[62]
France	2008	529	16.6	Genelabs ELISA	[63]
Switzerland	2011	550	4.9	MP Biomedical ELISA	[41]
Italy	2016	10 011	0.4	Wantai ELISA	[46]
Italy	2014	805	1.3	Dia.Pro ELISA	[64]
Italy	2014	3013	49	Wantai ELISA	[65]
Spain	2012	2305	2.7	Dia.Pro ELISA	[66]
Portugal	1998	50	4	Abbott ELISA	[67]
Serbia	2014	200	15	In-house ELISA	[39]
Poland	2015	3079	1.3	Wantai ELISA	[40]
Japan	2010	12 600	3.4	In-house ELISA	[68]
North Thailand	1996	663	8.7	Anogen ELISA	[69]
KSA Jeddah	1998	593	16.9	Bioelisa ELISA	[70]
KSA Makkah	2009	900	18.7	Bioelisa ELISA	[53]
Qatar	2017	5854	20.5	Qanti ELISA	[71]
India	2017	2447	0.2	In-house ELISA	[54]
New Zealand	2018	1013	9.7	Wantai ELISA	[51]
			8.1	MP Biomedical ELISA	
China	2014	10 741	27.42	Wantai ELISA	[72]

ELISA, enzyme-linked immunosorbent assay; DSI, Diagnostic Systems Incorporated; MP, MP Biomedicals.

**Table 3.** Rates of HEV RNA positivity among blood donors worldwide

Region	Year	Sample size	RNA ratio	Assay used	Reference
England	2011	42 000	1:7000	RT-PCR (mini plasma pool of 48 samples)	[73]
England	2014	22 500	1:2848	RT-PCR (mini plasma pool of 24 samples)	[16]
Scotland	2013	43 560	1: 14520	Nested PCR (mini plasma pool of 12 samples)	[35]
Ireland	2016	24 985	1:4997	Procleix TMA (individual samples)	[37]
Germany	2011	18 100	1:4525	RT-PCR (mini plasma pool of 96 samples)	[74]
Germany	2011	16 125	1:1241	RT-PCR (mini plasma pool of 48 samples)	[38]
Austria	2015	58 915	1:8416	RT-PCR (mini plasma pool of 96 samples)	[43]
The Netherlands	2011	45 415	1:2672	RT-PCR (mini plasma pool of 48 samples)	[32]
Sweden	2011	95 835	1:796	RT-PCR (mini plasma pool of 96 samples)	[74]
France	2012	53 234	1:2218	RT-PCR (mini plasma pool of 48 samples)	[29]
Southern France	2015	591	1:3353	RT-PCRa	[75]
Spain	2013	9998	1:3333	Procleix TMA (individual samples)	[44]
Serbia	2014	200	0	RT-PCR (individual samples)	[39]
Central Italy	2016	313	1:157	RT-PCR (mini plasma pool of 10 samples)	[65]
Italy	2018	10 011	0	Different methodological approaches b	[46]
Poland	2015	12 664	1:2109	TMA	[40]
Russia	2014	-	0	Real-time RT-PCR and nested PCR	[76]
US	2011	51 075	0	RT-PCR (mini plasma pool of 96 samples)	[74]
Brazil	2015	300	0	Nested PCR (HEV IgM-positive samples only)	[77]
Egypt	2011	760	1:375	RT-PCR (HEV IgM-positive samples only)	[78]
Qatar	2017	5854	1:1463	RT-PCR (HEV IgM-positive samples only)	[71]
Iran	2016	700	1:100	RT-PCR (HEV IgM/IgG-positive samples only)	[79]
Japan	2016	620 140	1:15,075	RT-PCR (mini plasma pool of 50 samples)	[80]
Thailand	2015	30155	1:1158	RT-PCR (mini plasma pool of 6 samples)	[81]
India	2017	2447	1:1223	Nested RT-PCR (HEV IgM-positive samples only)	[54]
Kashmir India	2004	145	1:27	Nested RT-PCR (individual samples)	[20]
Australia	2016	74 131	1:74,131	TMA (mini plasma pool of 6 samples)	[82]
Australia	2016	14 799	1:14,799	Procleix TMA (individual samples)	[83]
New Zealand	2018	5000	0	RT-PCR	[51]
China	2014	139	4:139	RT-PCR (HEV IgM/antigen-positive samples only)	[72]

a HEV RNA was tested in 99 HEV IgM-positive samples and 492 randomly selected IgM-negative specimens, independent of IgG status.

b The anti-HEV IgG-positive samples were tested by in-house PCR (mini plasma pool of 2–3 samples), and the anti-HEV IgG-negative samples were tested by RealStar RT-PCR (mini plasma pool of 10 samples); all of the anti-HEV IgM-positive samples were tested by RealStar RT-PCR (individual samples). TMA, transcription-mediated amplification.

Recently, a comprehensive meta-analysis was published that combined all anti-HEV IgG, IgM, HEV RNA and antigen-positive rates among Chinese blood donors from 22 independent studies [42]. The meta-analysis showed that the pooled positive rates of IgG, IgM, HEV RNA and antigen in Chinese blood donors were 29.2%, 1.1%, 0.1% and 0.1%, respectively, indicating that China has a high risk of TT-HEV infection, which remained steady from year to year. The anti-HEV IgG-positive rate differed depending on the province in China: it was higher in the South than the North, indicating that geographical region is an important factor affecting HEV infection, similar to previously published data from other countries [72]. As with the report in France in 2011 [84], this study did not observe a significant association between gender and prevalence of HEV infection, despite other studies showing a gender bias for males having higher anti-HEV IgG-positive rates [72]. Of note, the actual rate of HEV RNA positivity in the Chinese blood donors might have been an underestimate given that only those cases that were counted in this meta-analysis were from Chinese blood donors that were previously found to be positive for anti-HEV IgM or IgG antibodies which excluded those that did not score initially. This likelihood is underscored by a previous study from France in which 22 out of 24 cases from 53 234 blood donors that tested positive for HEV RNA were found negative for anti-HEV IgG and IgM [29].

## Worldwide blood donation HEV screening strategies

The screening of HEV RNA among blood donors is currently recognised as the only effective means to prevent TT-HEV infection. This is because risk factor assessment of blood donors before donation is not effective for HEV, since all donors are considered to be at risk due to dietary factors [36, 85], and existing blood virus inactivation techniques have been shown to be ineffective for HEV [13]. Ireland, the UK, France, the Netherlands, Germany, Spain, Austria, Luxembourg [26], Switzerland [86] and Japan [87] have all implemented screening protocols to deal with TT-HEV, with several other countries following suit [26]. Nevertheless, there remains a wide range of views on the necessity for government-mandated HEV donor screening programmes given the myriad factors impacting screening strategy, such as the prevalence of HEV in the region, cost–benefit of screening, health resource availability, etc. For example, after a cost–benefit analysis on the HEV blood donation screening strategy currently used in the Netherlands, researchers concluded that the cost of preventing TT-HEV through blood screening was not excessively high compared to other blood screening programmes; however, considering that only a small number of HEV infections are due to blood transfusions, the overall impact on preventing HEV spread would be small [88]. Meanwhile, in the USA and Canada, a similar study concluded that HEV blood screening would not be necessary because HEV prevalence in North America is far lower than in most other developed countries, meaning that the cost to implement would be expensive for little benefit [89]. A similar conclusion was reached for both Denmark and Sweden after evaluation of the prevalence of HEV RNA in their respective blood donation systems [74, 90].

### Universal *vs.* selective HEV screening

HEV screening strategies can be broadly classified into the following two types: universal screening of all blood donors (used in Ireland, the UK and the Netherlands) and selective screening (used in France, Austria and Luxembourg). A selective screening strategy refers to the screening of blood donation supplies only for patients that are deemed at a high risk of developing complications from TT-HEV. Although the selective screening strategy in principle may seem to be more cost-effective than a universal screening strategy due to its more limited use, in practice it can be more difficult to effectively implement. First, determining which patients constitute a ‘high risk’ for HEV infection is currently problematic given the lack of a standard of definition for what constitutes ‘high risk’. Conventionally, ‘high-risk’ patients were deemed those that were immunocompromised (transplant patients, HIV patients, cancer patients undergoing chemotherapy, etc.) in addition to pregnant women and the elderly; however, there is some debate as to whether to include patients with rheumatoid arthritis and other rheumatoid diseases who receive immunosuppressive drugs too. Second, blood transfusions are often needed during emergency situations, and on-demand screening for HEV in blood supplies for a ‘high-risk’ patient will likely be impossible to perform under such time constraints. Third, there is still a lot of debate as to whether HEV screening may be necessary even for immunocompetent patients. Although most immunocompetent patients that contract TT-HEV infections are typically subclinical in presentation, acute HEV infection and complications have been reported for some [91–93]. Fourth, selective screening often incurs increased cost logistics, as this creates two separate blood banks (HEV screened and unscreened) which necessitates greater manpower, material resources for classified storage and management, as well as increased risk of waste from letting unused supplies expire.

### ID-NAT *vs.* MP-NAT as HEV screening protocols

Currently, there is no universally adopted standard for choosing between individual donation (ID)-NAT or mini-pool (MP)-NAT protocols when screening for HEV in blood supplies, and thus practices vary widely across laboratories worldwide. While the sensitivity of ID-NAT is obviously higher than that of MP-NAT, the cost of implementation can make ID-NAT prohibitive for large-scale blood screening programmes. Even with the inherent trade-off of cost *vs.* sensitivity, the main issue for selecting a screening protocol is the lack of accurate data regarding the minimum virus load (VL) of which TT-HEV infection can occur [16, 26], and this can vary across different blood products and sources for a variety of reasons. For example, while all types of blood products can be a source for TT-HEV infections, Dreier *et al*. [94] recently concluded that having more plasma components carries a higher risk of transmission. Thus, products with lower plasma volume components (such as red blood cells and platelets) are less likely to transmit HEV compared to products with high plasma volume components (such as fresh frozen plasma). They also reported that the median infectious dose resulting in HEV infection was 520 000 IU, irrespective of the immune status of the recipient. When combined with data from Tedder *et al*. [85] that could not detect a TT-HEV infection in recipients from donor samples with >19 000 IU, this means that the minimum VL is likely between these extremes with the actual value tilting closer to that latter's estimates.

But perhaps the biggest hurdle towards achieving a reliable minimum VL for TT-HEV is that quantification of HEV RNA levels is subject to the detection limit of the analysis system being used. Two recent studies have published that the detection limits for two commonly used commercially available systems for measuring HEV RNA in serum, an MP-96 protocol using the RealStar RT-PCR HEV kit and the MP-24-based protocol using the Cobas HEV assay, were 452 IU/ml (95% LoD 4.7 IU/ml) and 446.4 IU/ml (95% LoD 18.6 IU/ml), for single donors, respectively [95, 96]. While generally impressive, for standard routine testing of blood banks, where detectable HEV viraemia in pooled sample testing is often considerably lower than from serum samples from patients with acute infection, this sensitivity might be inadequate. For example, multiple reports from HEV testing of blood donor samples out of Ireland showed that 59% of HEV-reactive samples had a VL of <450 IU/ml [26, 37, 97, 98], making them lower than the detection limits for either the RealStar or Cobas HEV assays, and thus would have been deemed as false-negative. Moreover, detection limits can vary even within the same sample, depending on whether ID-NAT or MP-NAT is used. Vollmer *et al*. [95] compared the results of MP-96-NAT with a detection limit of 447.4 IU/ml (95% LoD 4.66 IU/ml using the RealStar HEV RT-PCR assay) to ID-NAT (95% LoD 11.71 IU/ml using the Cobas HEV assay) in a German blood donor cohort. It was found that the rate of ID-NAT positivity was about 50% higher than that of MP-96-NAT. Nevertheless, the VL for most of the positive samples was lower than 25 IU/ml which would not normally cause TT-HEV, and thus, it was suggested that there was no added benefit to implement ID-NAT over MP-NAT-96 in screening German blood donation.

Finally, it should be noted that although the burden of disease from blood products with a low VL appears to be small (especially for immunocompetent patients), low-VL blood products can still lead to TT-HEV. A case from Germany [17] was reported where a possible TT-HEV derived infection that occurred in an immunocompetent patient who accepted an apheresis platelet transfusion from a single donor at levels that would have normally been deemed below the minimum VL (120 IU/ml HEV RNA/ml plasma; infectious dose of 8892 IU). This suggests that we should probably re-evaluate what the lower VL threshold is for TT-HEV in blood products for transfusion safety and disease burden overall, to better guide standard screening practices.

## Summary and outlook

As the global incidence of HE continues to rise, medical authorities are finally coming around to acknowledging the growing importance of HEV infection as a public health concern. In 2011, China's State Food and Drug Administration (SFDA) approved the first HEV-specific preventive vaccine. All of the clinical studies using Hecolin (HE vaccine produced in *Escherichia coli* (*E. coli*); Xiamen Innovax Biotech, China) were conducted in China, and the results showed that the vaccine was effective against genotypes 1 and 4 [99]. However, as of yet there are no clinical trial data regarding the effectiveness of Hecolin among high-risk populations nor of its effectiveness against type 3 HEV, the more common genotype among developed countries outside of Asia. Moreover, the World Health Organization's Global Advisory Committee on Vaccine Safety has recommended that a Phase IV post-marketing study should be carried out to further evaluate its safety before widespread implementation. While vaccination remains an important stalwart in combatting HEV infection more broadly, it would seem rather limited as an approach towards preventing the transmission of HEV among blood transfusion patients. Pathogen inactivation methods are considered to be the best means towards eliminating viral contamination of blood products, but current techniques are ineffective for HEV. Until newer blood treatment techniques are developed, there appears to be no better way to prevent TT-HEV infection other than by screening for HEV in blood donation products. It is perhaps not surprising that global experts disagree on the necessity and urgency of introducing HEV screening in blood donors, given its complexity in implementation, cost–benefit considerations and few large-scale studies. However, the overarching reason that limits widespread adoption of HEV screening in blood donation is how rare TT-HEV infections are among patients. First, the spread of HEV is far more likely to have happened from a contaminated food or water source than from blood transfusion. Second, reports of TT-HEV infections remain scarce in the literature. This, however, is likely a gross underestimation of incidence, given the often asymptomatic outcome of infection and the lack of routine HEV screening, which, collectively, reinforce the assumption of the rarity of TT-HEV infections.

Going forward, the primary solution towards preventing HE will be to monitor and break the faecal–oral transmission route of HEV transmission, a laborious task that will require a campaign of greater awareness among the general population. Secondarily to that, food inspection should be enhanced, particularly during processing, packaging and preparation, e.g. through the promotion of hygienic diets (limiting raw meat), controlling animal farm waste runoff into irrigation water reservoirs, etc. At the same time, the implementation of HEV blood screening is an effective and feasible preventive measure, albeit its cost-effectiveness will need to be assessed on a regional and population-specific basis. The hope is that the near future will bring the continued development of more effective vaccines and improved pathogen inactivation technologies that will radically alter how we manage and constrain the spread of HEV infection.

## Data Availability

All data described in this review are from previously published papers and available from the cited references
